# Mathematical modeling and topological graph description of dominating David derived networks based on edge partitions

**DOI:** 10.1038/s41598-023-42340-6

**Published:** 2023-09-13

**Authors:** Shahid Zaman, Wakeel Ahmed, Atash Sakeena, Kavi Bahri Rasool, Mamo Abebe Ashebo

**Affiliations:** 1 Department of Mathematics, University of Sialkot, Sialkot, 51310 Pakistan; 2 Faculity of Science, University of Zakho, Duhok, Kurdistan Region Iraq; 3 Department of Mathematics, Wollega University, 395 Nekemte, Ethiopia

**Keywords:** Mathematics and computing, Applied mathematics, Computational science

## Abstract

Chemical graph theory is a well-established discipline within chemistry that employs discrete mathematics to represent the physical and biological characteristics of chemical substances. In the realm of chemical compounds, graph theory-based topological indices are commonly employed to depict their geometric structure. The main aim of this paper is to investigate the degree-based topological indices of dominating David derived networks (DDDN) and assess their effectiveness. DDDNs are widely used in analyzing the structural and functional characteristics of complex networks in various fields such as biology, social sciences, and computer science. We considered the F_N_^*^, ${M}_{2}^{*}$, and ${HM}_{N}$ topological indices for DDDNs. Our computations' findings provide a clear understanding of the topology of networks that have received limited study. These computed indices exhibit a high level of accuracy when applied to the investigation of QSPRs and QSARs, as they demonstrate the strongest correlation with the acentric factor and entropy.

## Introduction

In the field of graph theory, specifically in chemical graph theory, a chemical molecule is represented by a molecular graph, which is a simple graph. In this representation, vertices denote the atoms and edges represent bonds or connections. The edges goes beyond simple connectivity; it encompasses the type of bond as well. For instance, a single edge might denote a single covalent bond, while a double edge could represent a double bond involving the sharing of two pairs of electrons.

The emerging field of cheminformatics, which explores the relationship between quantitative structure–activity and structure–property, is gaining momentum as it aids in the prediction of biological activities. Topological indices are important invariants derived from graph theory that enable the characterization of a graph's topology. A topological index is a numerical value that provides information about the structure of a graph. Topological indices help in identifying various characteristics of a graph. Furthermore, the topology of a graph remains invariant under the automorphisms of graphs. Comperisons of the degree based toplogical indices hold a particularly significant place in research^1, 2^.

The first toplogical index introduced by Wiener, during the research of paraffin melting point. Initially termed “path number”, it was later renamed and has since become known as the Wiener index. Researchers have put a lot of effort into studying chemical graph theory. A key component of graph theory's work involves honeycomb networks. The honeycomb shape, with its hexagonal pattern of cells, finds a wide range of applications across various fields due to its unique structural and geometric properties. Some of the notable applications of the honeycomb shape include as: In structural engineering and architecture the honeycomb structure's hexagonal arrangement provides exceptional strength and stability while using minimal material. This makes it suitable for applications in construction, such as in lightweight yet strong support structures, building facades, and panels. On the other hands, in art and design, the visually appealing hexagonal pattern of the honeycomb has inspired artists, designers, and architects to incorporate it into their creations. From decorative elements in interior design to art installations, the honeycomb pattern adds a unique aesthetic.

In this article the notation E denotes an edge set and V denotes the vertex set of a graph G. The expression $\eta_{G} \left( v \right)$ is the number of edges overall connected to a particular vertex v.

For the sake of simplicity, assume that a and b are two adjacent vertices and E is an edge between them, then the edge partition of E is denoted by ${E}_{a,b}$ and formulated as $E_{a,b} = \{ \eta_{G} (a)\,,\,\,\eta_{G} (b)\}$.

The degree-based topological indices shows a significant role in the field of mathematical chemistry^3–7^, and widely used to develop models that accurately predict the boiling points of alkanes with carbon atom^8^. Some current discovered degree-based neighborhood indices are presented in^9, 10^ and shown strong connections between entropy and the acentric factor.

In^11–16^, different chemical significant graphs' topological indices are considered. Baig et al.^17^ considered the topological indices for several silicates and oxide networks. Ullah et al.^18^, compared and examined the computational characteristics of two carbon nanosheets using some innovative topological indices. The topological characteristics of rhombus-type silicate and oxide networks were explored by Javaid et al.^19^. Recently, Koam et al.^20^, established the entropy measures of Y-junction based nanostructures. Ali et al.^21^ give some properties of ve-degree based topological indices for hex-derived networks. In this study, an examination was conducted on distance-based topological polynomials that are associated with zero-divisor graphs, as discussed in^22^. The authors of^23^ obtained the polynomials of degree-based indices of metal–organic networks. Zaman et al., determined the kemeny’s constant and spanning trees of hexagonal ring network^24^. Some upper bound and lower bound of graphs and also the spectral analysis of graphs are discussed in^25–28^. In this research, inspired by earlier studies, we establish some exact expressions of the different types of Dominating David derived networks and their comparisons.

We have calculated the forgotten index ($F_{N}^{ * }$)^29^, the second zargeb index ($M_{2}^{ * }$)^30^ and the Harmonic index (${HM}_{N}$)^31^ for DDD networks. These topological indices are defined as $F_{N}^{ * } = \sum\limits_{uv \in E\left( G \right)} {\left[ {\eta_{G} \left( u \right)^{2} + \eta_{G} \left( v \right)^{2} } \right]}$, $HM_{N} \left( G \right) = \sum\limits_{uv \in E\left( G \right)} {\left[ {\eta_{G} \left( u \right) + \eta_{G} \left( v \right)} \right]}^{2}$, $M_{2}^{ * } = \sum\limits_{uv \in E\left( G \right)} {\left[ {\eta_{G} \left( u \right) + \eta_{G} \left( v \right)} \right]}$.

## Constructions of dominating David derived networks (DDDN)

**Figure 1:**
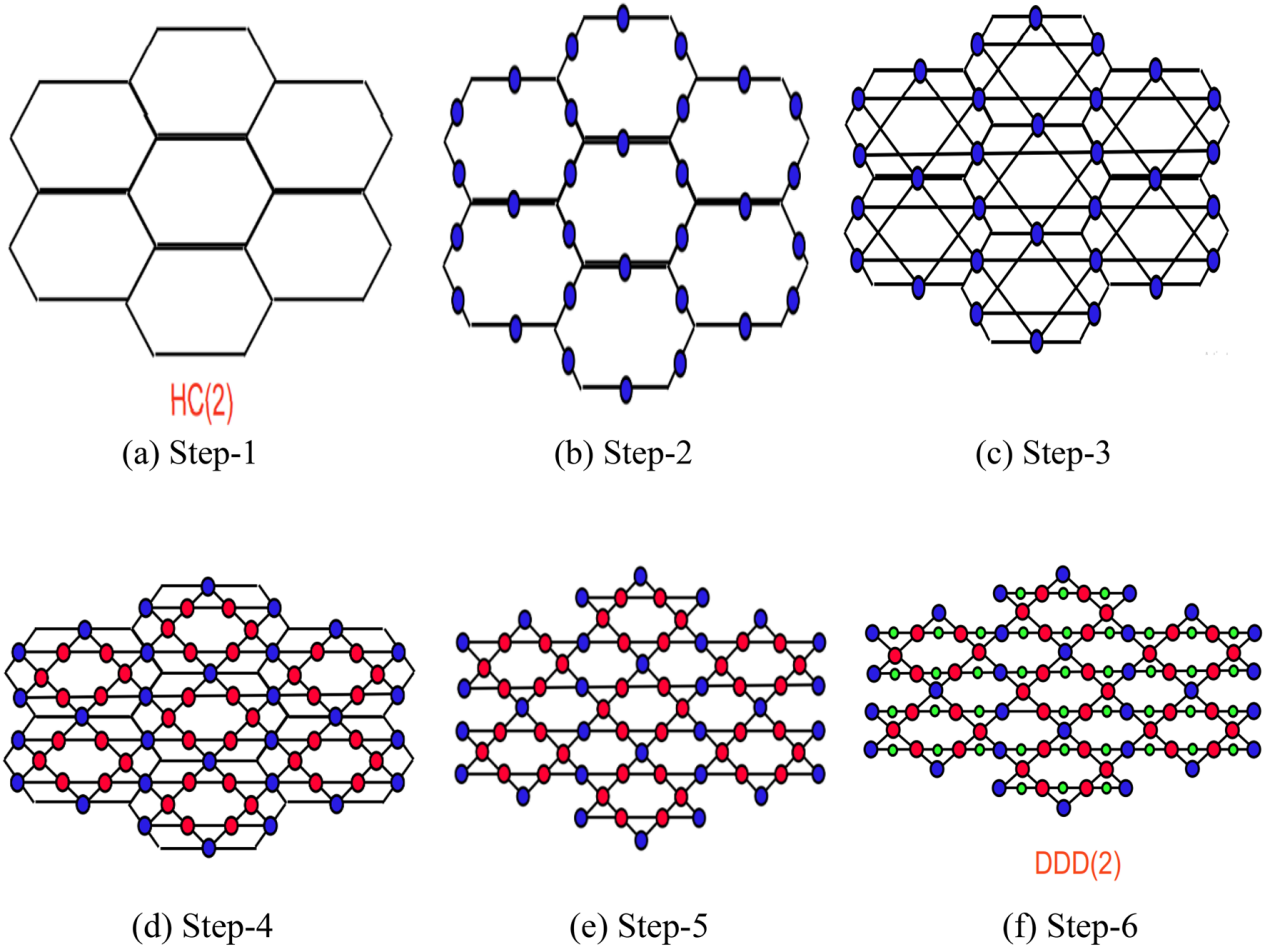
The steps to derive DDD (2).

In the field of chemistry, honeycomb networks are utilized as representations for benzoid hydrocarbons. Honeycomb networks find extensive applications in various domains, including graphics, such as cell phone base stations and image processing. The honeycomb network is formed by enclosing the boundaries with a layer of hexagons. Based on the honeycomb network, different types of Dominating David derived networks can be derived. One can follow the below steps to construct the DDDN (t dimension):

Step 1: Consider a t-dimension honeycomb network (see Fig. 1a).
Step 2: Add another vertex to divide each edge into two pieces (see Fig. 1b).
Step 3: In each hexagonal cell, connect the new vertices by an edge if they are at a distance of 4 within a hexagon (see Fig. 1c).
Step 4: Add new vertices at new edge intersections. (see Fig. 1d).
Step 5: Remove the starting vertices and edges of the honeycomb (see Fig. 1e).
Step 6: Divide each horizontal edge into two parts by addind a new vertex (see Fig. 1f).

## Main results

**Figure 2:**
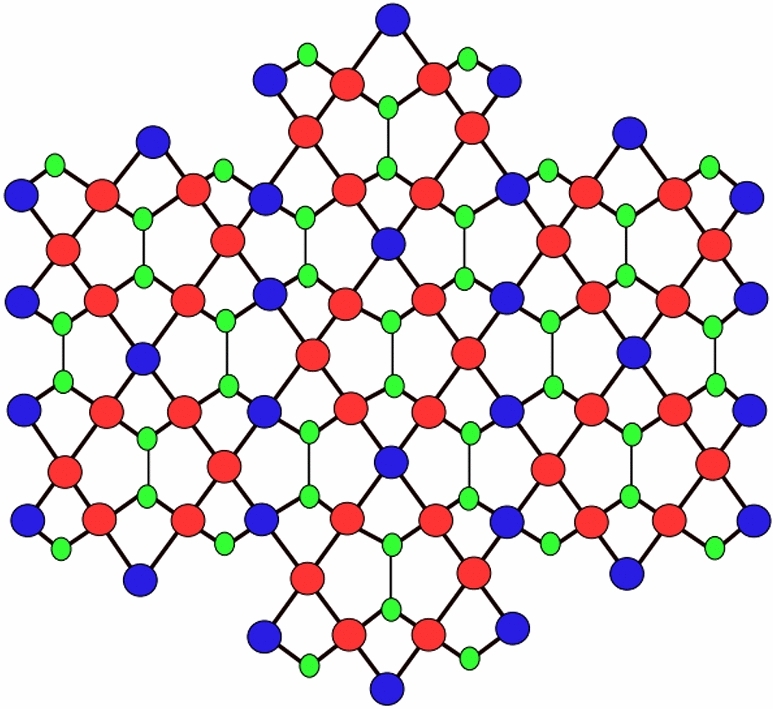
First type of D_1_(2) network.

**Figure 3:**
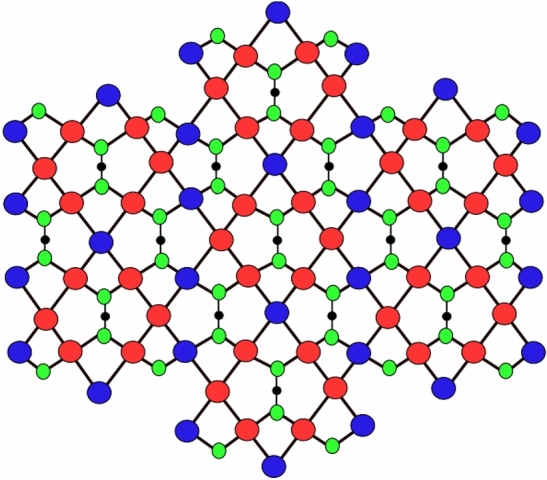
First type of D_2_(2) network.

**Figure 4:**
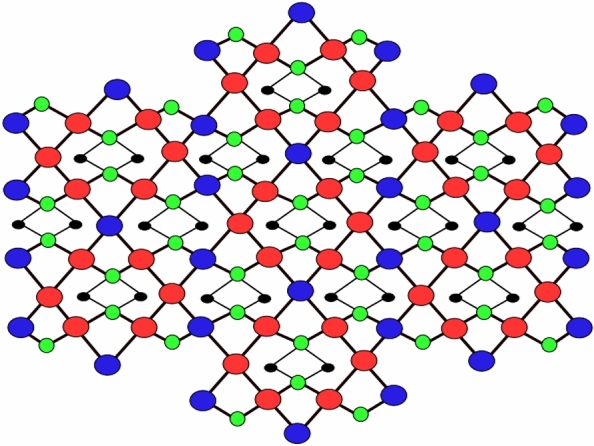
Third type of D_3_(2) network.

Our key findings rely on the edge partitions of Figs. 2, 3 and 4 as given below. We have calculated these edge partitions based on the degrees of the end vertices of each edge. For instance, the first row of Fig. 1 shows the degrees of the end vertices of edges, while the second row illustrates the count of edges with those specific degrees. In the same way, we have obtained the other tables.

### The F_N_^*^ topological index for dominating David derived networks

Let G be a graph in D_1_(t), D_2_(t) and D_3_(t) then according to the definition of $F_{N}^{ * }$ and Table 1, we have

$$\begin{aligned} F_{N}^{ * } \left( G \right) & = \sum\limits_{{uv \in {\rm E}\left( G \right)}} {\left[ {\eta_{G} \left( u \right)^{2} + \eta_{G} \left( v \right)^{2} } \right]} \\ & = \left| {{\rm E}_{{\left( {2,2} \right)}} } \right|\left[ {\left( 2 \right)^{2} + \left( 2 \right)^{2} } \right] + \left| {{\rm E}_{{\left( {2,3} \right)}} } \right|\left[ {\left( 2 \right)^{2} + \left( 3 \right)^{2} } \right] + \left| {{\rm E}_{{\left( {2,4} \right)}} } \right|\left[ {\left( 2 \right)^{2} + \left( 4 \right)^{2} } \right] + \left| {{\rm E}_{{\left( {3,3} \right)}} } \right|\left[ {\left( 3 \right)^{2} + \left( 3 \right)^{2} } \right] \\ & \quad + \left| {{\rm E}_{{\left( {3,4} \right)}} } \right|\left[ {\left( 3 \right)^{2} + \left( 4 \right)^{2} } \right] \\ & = 4t\left( 8 \right) + 4t - 4\left( {13} \right) + 28t - 16\left( {20} \right) + 9t^{2} - 13t + 5\left( {18} \right) + 36^{2} - 56t + 24\left( {25} \right) + 36t^{2} - 52t + 20\left( {32} \right) \\ & = 32t + 52t - 52 + 560t - 320 + 162t^{2} - 234t + 90 + 900t^{2} - 1400t + 600 + 1152t^{2} - 1664t + 640 \\ & = 2214t^{2} - 1951t + 958 \\ \end{aligned}$$

**Table 1:** Edges partition D_1_(t).

$(\eta_{G} (u)\,,\,\,\eta_{G} (v))$	$\left( {2,2} \right)$	$\left( {2,3} \right)$	$\left( {2,4} \right)$	$\left( {3,3} \right)$	$\left( {3,4} \right)$	$\left( {4,4} \right)$
Frequency	$4t$	$4t - 4$	$28t - 16$	$9t^{2} - 13t + 5$	$36t^{2} - 56t + 24$	$36t^{2} - 52t + 20$

Similarly, from Table 2, we have

$$\begin{aligned} F_{N}^{ * } \left( G \right) & = 32t + 234t^{2} - 286t + 78 + 560t - 320 + 900t^{2} - 1400t + 600 + 1152t^{2} - 1664t + 640 \\ & = 2286t^{2} - 2758t + 998 \\ \end{aligned}$$

**Table 2:** Edge partition D_2_(t).

$(\eta_{G} (u)\,,\,\,\eta_{G} (v))$	$\left( {2,2} \right)$	$\left( {2,3} \right)$	$\left( {2,4} \right)$	$\left( {3,4} \right)$	$\left( {4,4} \right)$
Frequency	$4t$	$18t^{2} - 22t + 6$	$28t - 16$	$36t^{2} - 56t + 24$	$36t^{2} - 52t + 20$

And from Table 3, one has

$$\begin{aligned} F_{N}^{ * } \left( G \right) & = \left| {E_{(2,2)} } \right| \, \left( {4 + 4} \right) + \, \left| {E_{(2,4)} } \right| \, \left( {4 + 16} \right) + \, \left| {E_{(4,4)} } \right| \, \left( {16 + 16} \right) \\ & = 32t + 720t^{2} - 400t + 2304t^{2} - 3456t + 1408 \\ & = 3024t^{2} - 3824t + 1408 \\ \end{aligned}$$

**Table 3:** Edge partition D_3_(t).

$(\eta_{G} (u)\,,\,\,\eta_{G} (v))$	$\left( {2,2} \right)$	$\left( {2,4} \right)$	$\left( {4,4} \right)$
Frequency	$4t$	$36t^{2} - 20t$	$72t^{2} - 108t + 44$

### The ${{\varvec{M}}}_{2}^{\boldsymbol{*}}$ topological index for DDDN

Let G be a graph in D_1_(t), D_2_(t) and D_3_(t) then according to the definition of ${M}_{2}^{*}$ and Table 1 we have

$$\begin{aligned} M_{2}^{*} (G) & = E_{(2,2)} \left| { \, \left( {2.2} \right) \, + \, } \right|E_{(2,3)} \left| { \, \left( {2.3} \right) + \, } \right|E_{(2,4)} \left| { \, \left( {2.4} \right) + \, } \right|E_{(3,3)} \left| { \, \left( {3.3} \right) \, + \, } \right|E_{(3,4)} \left| { \, \left( {3.4} \right) + \, } \right|E_{(4,4)} | \, \left( {4.4} \right) \\ & = 16t + 24t - 24 + 224t - 128 + 81t^{2} - 117t + 45 + 432t^{2} - 672t + 288 + 576t^{2} - 832t + 320 \\ & = 1089t^{2} - 1357t + 501 \\ \end{aligned}$$

Likewise, based on the information presented in Table 2, we obtain

$$\begin{aligned} M_{2}^{*} (G) & = 16t + 108t^{2} - 132t + 36 + 224t - 128 + 432t^{2} - 672t + 288 + 576t^{2} - 832t + 320 \\ & = 1116t^{2} - 1396t + 516 \\ \end{aligned}$$

And from Table 3, one has

$$\begin{aligned} M_{2}^{*} (G) & = \left| {E_{(2,2)} } \right|\left( {2.2} \right) + \left| {E_{(2,4)} } \right|\left( {2.4} \right) + \left| {E_{(4,4)} } \right|\left( {4.4} \right) \\ & = 16t + 288t^{2} - 160t + 1152t^{2} - 1728t + 704 \\ & = 1440t^{2} - 1872 + 704 \\ \end{aligned}$$

### The ${{\varvec{H}}{\varvec{M}}}_{{\varvec{N}}}$ topological index for DDDN

Let G be a graph in D_1_(t), D_2_(t) and D_3_(t) then according to the definition of ${HM}_{N}$ and Table 1, we have

$$\begin{aligned} & HM_{N} \left( G \right) = 64t + 100t - 100 + 1008t - 576 + 324t^{2} - 468t + 180 + 1764t^{2} - 2744t + 1176 + 2304t^{2} - 3328t + 1280 \\ & HM_{N} \left( G \right) = 4392t^{2} - 5362t + 3240 \\ \end{aligned}$$Similarly, from Table 2, we have

$$\begin{aligned} HM_{N} \left( G \right) & = 64t + 450t^{2} - 55t + 150 + 1008t - 576 + 1764t^{2} - 2744t + 1176 + 2304t^{2} - 3328t + 1280 \\ HM_{N} \left( G \right) & = 4518t^{2} - 5055t + 2030 \\ \end{aligned}$$

And from Table 3, we have

$$\begin{aligned} HM_{N} \left( G \right) & = \left| {E_{(2,2)} } \right|\left( {16} \right) + \left| {E_{(2,4)} } \right|\left( {36} \right) + \left| {E_{(4,4)} } \right|\left( {64} \right) \\ & = 64t + 1296t^{2} - 720t + 4608t^{2} - 6912t + 2816 \\ & = 5904t^{2} - 7568t + 2816 \\ \end{aligned}$$

## Concluding Remarks

In this study, we have considered the ${F}_{N}^{*}$, ${M}_{2}^{*}$ and $HM_{N}$ topological indices. Our simulated results help for the better comprehend topology and enhance physical properties of the honeycomb structure. The computed indices, and above, as previously mentioned, have the most closely relates to the acentric factor and entropy consequently, they are extremely accurate in QSPR and QSAR analysis.

**Figure 5:**
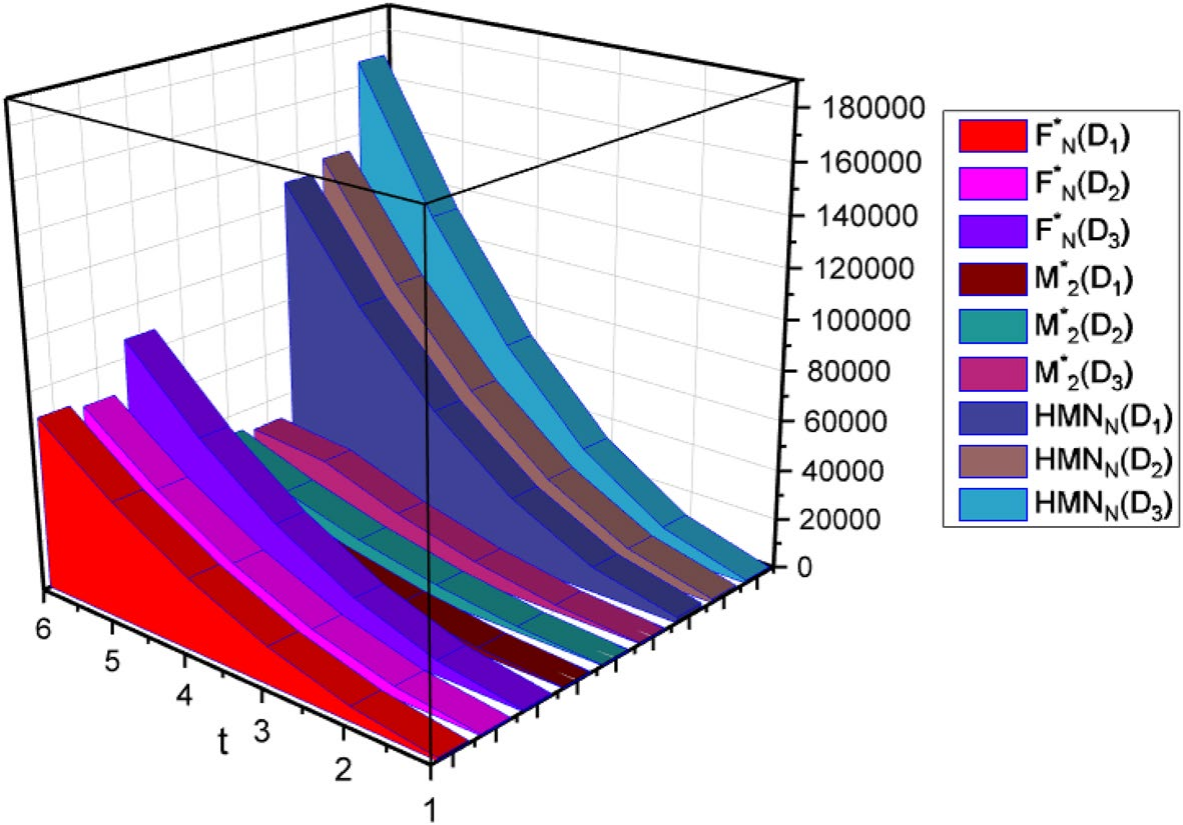
The comparison graph.

In Table 4, the topological indices computed are represented mathematically. As we can see, increasing the values of t, increases the value of the indices as well. We have precise analytical formulations for the D_1_, D_2_ and D_3_ networks, considering various topological indices. In the rapidly expanding fields of nanotechnology and applications, such as networks, our current discoveries and techniques can be applied to other, more complex structures. The utilization of distance-based topological indices poses greater challenges and complexity, but they can be employed alongside existing methods. Exploring these types of studies will be the focus of future research endeavors. In Table 4 and Fig. 5, we computed the numerical comparison of the certain topological indices for D_1_, D_2_ and D_3_ networks, which shows that when we increase t as a result the values of the topological indices also increases. These numerical comaprisons also shows that the inceasing rate of $HM_{N}$ for D_3_ is greater than the other topological indices. Since, in graph theory, the $HM_{N}$ is a mathematical concept used to describe the connectivity. Therefore, a higher $HM_{N}$ reflects the more connectivity among the atoms of a molecule. This indicates that the D_3_ molecule has a greater potential for forming diverse interactions with other molecules and participating in a wider range of chemical reactions.

**Table 4:** The comparison of ${F}_{N}^{*}$, ${M}_{2}^{*}$ and $HM_{N}$ for D_1_(t), D_2_(t) and D_3_(t) graphs.

$t$	${F}_{N}^{*}$(D_1_)	${F}_{N}^{*}$(D_2_)	${F}_{N}^{*}$(D_3_)	${M}_{2}^{*}$(D_1_)	${M}_{2}^{*}$(D_2_)	${M}_{2}^{*}$(D_3_)	$HM_{N}$(D_1_)	$HM_{N}$(D_2_)	$HM_{N}$(D_3_)
1	1221	526	608	233	236	272	2270	1493	1152
2	5912	4626	5856	2143	2188	2720	10,084	9992	11,296
3	15,031	13,298	17,152	6231	6372	8048	26,682	27,527	33,248
4	28,578	26,542	34,496	12,497	12,788	16,256	52,064	54,098	67,008
5	46,553	44,358	57,888	20,941	21,436	27,344	86,230	89,705	112,576
6	68,956	66,746	87,328	31,563	32,316	30,512	129,180	134,348	169,952

## Data Availability

All data generated or analysed during this study are included in this published article.

## References

[1] Zaman S (2023). Mathematical analysis and molecular descriptors of two novel metal–organic models with chemical applications. Sci. Rep..

[2] Liu H (2023). Comparison between Merrifield–Simmons index and some vertex-degree-based topological indices. Comput. Appl. Math..

[3] Babar U (2020). Multiplicative topological properties of graphs derived from honeycomb structure. Aims Math..

[4] Ali H (2019). On the degree-based topological indices of some derived networks. Mathematics.

[5] Yang S (2022). Structure of Fejes Tóth cells in natural honey bee combs. Apidologie.

[6] Hayat S, Malik MA, Imran M (2015). Computing topological indices of honeycomb derived networks. Rom. J. Inf. Sci. Technol..

[7] Imran M (2016). On topological properties of poly honeycomb networks. Period. Math. Hung..

[8] Mukwembi S, Nyabadza F (2021). A new model for predicting boiling points of alkanes. Sci. Rep..

[9] Ahmad MS (2017). Calculating degree-based topological indices of dominating David derived networks. Open Phys..

[10] Imran M, Baig AQ, Ali H (2016). On topological properties of dominating David derived networks. Can. J. Chem..

[11] Aslam A (2017). On topological indices of certain dendrimer structures. Z. Naturforsch. A.

[12] Ullah A, Zeb A, Zaman S (2022). A new perspective on the modeling and topological characterization of H-naphtalenic nanosheets with applications. J. Mol. Model..

[13] Wang G (2020). The connective eccentricity index of graphs and its applications to octane isomers and benzenoid hydrocarbons. Int. J. Quantum Chem..

[14] Mondal S, De N, Pal A (2019). Topological properties of graphene using some novel neighborhood degree-based topological indices. Int. J. Math. Ind..

[15] De N (2020). On some degree based topological indices of mk-graph. J. Discrete Math. Sci. Cryptogr..

[16] Zaman S, Ali A (2021). On connected graphs having the maximum connective eccentricity index. J. Appl. Math. Comput..

[17] Baig AQ, Imran M, Ali H (2015). On topological indices of poly oxide, poly silicate, DOX, and DSL networks. Can. J. Chem..

[18] Ullah A (2022). Computational and comparative aspects of two carbon nanosheets with respect to some novel topological indices. Ain Shams Eng. J..

[19] Javaid M, Rehman MU, Cao J (2017). Topological indices of rhombus type silicate and oxide networks. Can. J. Chem..

[20] Koam AN (1913). Entropy measures of Y-junction based nanostructures. Ain Shams Eng. J..

[21] Ahmad A, Imran MJC (2022). Vertex-edge-degree-based topological properties for hex-derived networks. Complexity.

[22] Ahmad A (2022). Analysis of distance-based topological polynomials associated with zero-divisor graphs. Comput. Mater. Contin..

[23] Ahmad A (2022). Polynomials of degree-based indices of metal-organic networks. Comb. Chem. High Throughput Screen..

[24] Zaman, S., et al., *The Kemeny’s Constant and Spanning Trees of Hexagonal Ring Network.*

[25] Khabyah AA (2022). Minimum zagreb eccentricity indices of two-mode network with applications in boiling point and benzenoid hydrocarbons. Mathematics.

[26] Zaman S (2021). Maximum H-index of bipartite network with some given parameters. AIMS Math..

[27] Zaman S (2021). Cacti with maximal general sum-connectivity index. J. Appl. Math. Comput..

[28] Zaman S (2022). Spectral analysis of three invariants associated to random walks on rounded networks with 2 n-pentagons. Int. J. Comput. Math..

[29] Gao W (2016). Forgotten topological index of chemical structure in drugs. Saudi Pharm. J..

[30] Khalifeh M, Yousefi-Azari H, Ashrafi AR (2009). The first and second zagreb indices of some graph operations. Discrete Appl. Math..

[31] Zhong L (2012). The harmonic index for graphs. Appl. Math. Lett..

